# Uncertainty in Relation to Clinical Experience—A Qualitative Study on Dental Students' Reflections on Risk Assessment of Root Filled Teeth

**DOI:** 10.1111/iej.70047

**Published:** 2025-10-02

**Authors:** Joséphine Brodén, Maria Pigg, Niklas Vareman, Helena Fransson

**Affiliations:** ^1^ Department of Dental Medicine Karolinska Institutet Huddinge Sweden; ^2^ Department of Oral Biology, Faculty of Odontology Malmö University Malmö Sweden; ^3^ Department of Endodontics, Faculty of Odontology Malmö University Malmö Sweden; ^4^ Department of Medical Ethics Lund University Lund Sweden

**Keywords:** [clinical] decision‐making, dental education, endodontics, periapical periodontitis, uncertainty

## Abstract

**Aim:**

In uncertain clinical situations, such as assessing the risk of exacerbation of apical periodontitis in root‐filled teeth, undergraduate students can benefit from being trained to reflect. This study aimed to explore how students specifically reflected on clinical experiences in relation to uncertainty when assessing a case involving a root‐filled tooth with apical periodontitis.

**Methodology:**

Short reflections were written by 52 dental students at Malmö University in 2021. All students were asked to reflect on the risk for exacerbation of apical periodontitis in a case with a root‐filled tooth with a diffuse widening of the periodontal ligament space. The reflections were written according to prompts developed to stimulate reflection. For each student, the reflections were analysed by a qualitative method, Systematic Text Condensation (STC).

**Results:**

The analysis of the qualitative data resulted in the formulation of two themes about experience and lack of experience: Theme 1 ‘The meaning of clinical experience’ and Theme 2 ‘Differences and similarities’. The themes were subdivided into nine subgroups and described the relationship between experience and certainty.

**Conclusions:**

The students believed that certainty comes with experience even when there is a lack of scientific evidence.

## 1 Introduction

Future dentists need to develop an ‘adaptive expertise’ meaning that when facing new uncertain problems, they should be able to apply their knowledge from past experience as well as create new knowledge going forward (Mylopoulos et al. 2018). One way of preparing for these demands is to train students to engage in reflection (Schön 1987). Reflection is defined by Ryan and Ryan (2012) as ‘making sense of experience’ and ‘reimagining future experience’. Students can be trained in deep reflection by reporting on uncertain issues and making observations, asking questions, analysing, drawing conclusions and planning for future actions (Ryan and Ryan 2012).

A dentist can experience uncertainty on several different levels, for example, when assessing the risk for exacerbation of root‐filled teeth with persistent apical periodontitis. Qualitative studies involving dentists in private practice, public dental health and hospital settings identified uncertainty associated with the decision‐making process in such cases (Lindström et al. 2025; Olsson et al. 2023). In a previous study with the objective of increasing dental students' awareness of and comfort with uncertainty when assessing this risk, most students stated that while the students themselves felt uncertain, they thought that an experienced colleague would feel certain (Brodén et al. 2025). It is difficult for a student or an inexperienced clinician to know where the uncertainty lies, whether it is due to a personal lack of knowledge or to a more general lack of knowledge in the field (Fox 1957). There are several sources of scientifically related uncertainty related to the assessment. For example, the likelihood that an individual should develop an exacerbation or minor symptoms is unknown (probability), and science does not provide sufficient understanding of the causes of exacerbation (complexity). Furthermore, the threshold between what is healthy and what is disease associated with root‐filled teeth is not well‐defined (ambiguity). Because of this, it is impossible for anyone to actually *be* certain when assessing this risk (Pigg et al. 2022). Clinical experience is one of the cornerstones in evidence‐based practice along with the best scientific evidence and the patient's values and preferences (Shuler and von Bergmann 2021). Relying on experience in uncertain conditions is common in clinical decision making (Thompson 2003). It is not studied why dental students believe that an expert would feel certain in uncertain circumstances.

The aim of this study was to explore how dental students reflected on clinical experience in relation to uncertainty when assessing the risk for exacerbation of apical periodontitis in a case with a root filled tooth.

## 2 Material and Method

### 2.1 Context and Study Design

This study was a qualitative, cross‐sectional study based on written reflections by 52 students attending their fifth and final year of dental studies (undergraduate level) at Malmö University in Sweden in 2021. During their 3rd and 4th years, the students had completed their endodontic courses, the curricula of which covered no learning objectives regarding tolerance of uncertainty. The students had no training in structured reflection, although verbal reflection in study groups was an integrated part of the problem‐based learning curricula (Rohlin et al. 1998). Prior to conducting the study, the Swedish Ethical Review Authority was consulted and ruled that an ethical review was not necessary for this study (Dnr: 2019‐02441) since no personal data was collected.

### 2.2 Educational Activities

A reflection exercise was developed with questions and prompts for reflection derived from the 4Rs (Report & respond, Relate, Reason and Reconstruct), a framework by Ryan and Ryan (2012) adapted from the 5Rs by Bain et al. (2002) constructed to stimulate increasing levels of reflection. The prompts were pilot tested prior to publishing them on the online platform. The pilot involved two junior colleagues working in the endodontic department who gave feedback on the clarity of the prompts and wrote reflections (not included in the analysis). The educational activities were carried out on the online learning platform Canvas (Instructure Inc., Salt Lake City, UT, USA) in January and June 2021. As part of a separate study examining the impact of the exercise on students' comfort with uncertainty, the students were asked to complete a questionnaire about uncertainty and comfort with uncertainty before and after writing the reflections (Brodén et al. 2025). The participants were randomly assigned to one of two groups, completing the reflection exercise either in January or in June. The randomisation was not related to the aim of the current study, and no comparison between groups was thus made in the qualitative analysis reported in this paper.

During the exercise, the students were asked to reflect on a described case involving a patient with a symptom‐free root‐filled molar tooth with an intraoral radiograph showing a diffuse widening of the periodontal ligament space and a composite restoration and to write anonymous short reflections about the risk for exacerbation of apical periodontitis using prompts (Table 1).

**TABLE 1:** Questions and prompts adapted from a reflective scale by Ryan and Ryan (2012) used for the written reflections in educational activity. Translated from Swedish.

1	What is the case about? Describe the case from a biological point of view.
2	What strategy do you have when assessing the probability for exacerbation of apical periodontitis in this case?
3	What knowledge do you have about assessing the probability of exacerbation of apical periodontitis in a case like this? Think of a similar case that you have read about or encountered before. Is there any factor that is different in this case that could indicate a different biological condition? Explain!
4	What are the risks for the patient? What do you really know about the risk? What do you need to know more about? Explain!
5	Think through the biological process, based on theory, and fill in keywords describing biological factors from a leaking restoration to the exacerbation of apical periodontitis.
6	How do you think an expert would reason about the patient's condition and assess the risk? Is it different from your way of reasoning?
7	If your chosen strategy turns out to be wrong—it could mean either that you overtreated the patient, i.e., that you treated a condition that did not require treatment, or that you neglected to treat a condition that required treatment, i.e., undertreatment. Think about what is best from the perspective of efficient utilisation of resources and from the ethical perspective. Reflect on what could make you change strategy.

### 2.3 Data Collection

Data for the qualitative analysis was collected from these written reflections. Written information about the study was sent to the students 1 week prior to the educational activities and again on the day of the activities prior to writing the reflections. The mandatory educational activities involved 53 students. One student participated in the educational activities but declined to participate in the study, and their written reflections were excluded. The age of the participating 38 women and 14 men ranged from 23 to 41 years (mean 28.0 ± SD 4.9). The students were informed that participating in the study was voluntary and that the results would be used for research and quality assessment to ensure that dental students throughout their education are provided with the tools they need to deal with uncertainty.

### 2.4 Text Analysis and Researchers' Reflexivity

Four researchers were involved in the analysis of the text material. Three researchers (J.B., M.P. and H.F.) are dentists specialised in endodontology, and two (M.P. and H.F.) are senior lecturers who were involved in the teaching of the students participating in this study. One researcher (H.F.) had prior experience of qualitative research. One researcher (N.V.) is a PhD and an associate researcher in medical ethics. The researchers' preconceptions when adapting the prompts for the reflection exercise were that an expert would feel as uncertain about assessing the risk for exacerbation of apical periodontitis as a student since there is a lack of scientific evidence about assessing this risk (Pigg et al. 2022).

The content of the written reflections was analysed by Systematic Text Condensation (STC), in which Malterud (2012) has elaborated phenomenological methods into a new method that includes four steps (Table 2).

**TABLE 2:** The four steps of Systematic Text Condensation (STC) (Malterud 2012) and the authors involved in each step.

Steps according to STC	Involved authors
Reading through all text to get an overall impression and identifying preliminary themes.	J.B., M.P., H.F. and N.V.
2 Identifying and coding meaning units.	J.B.
3 Sorting the meaning units into subgroups. Reducing the content into a condensate (an artificial quotation from the merged different meaning units).	J.B. and H.F.
4 Describing categories from the condensates and illustrating with real citations.	J.B., M.P., H.F. and N.V.

After reading through all the reflections in the first step and creating preliminary themes around the initially intended research question (Table 3), it became clear that the reflections around the first five prompts (Table 1) mostly constituted common management strategies such as checklists of all further dental examinations that the students would like to undertake to reduce uncertainty. The students did also acknowledge the difficulty of assessing the risk in the individual case, the lack of scientific knowledge considering the risk of exacerbation of apical periodontitis, and the difficulty of taking health economic considerations in such an uncertain case. However, the reflections about experience and lack of experience caught the attention of the researchers since they appeared to suggest a pattern that could potentially add some new information to the field. The subsequent analysis therefore focused entirely on experience, and only the preliminary themes around experience were further explored (Table 3).

**TABLE 3:** The process from initial research question to research question about experience.

Steps of the process	Research questions and themes
Initial research question	How did the dental students express uncertainty and its consequences in this reflection exercise?
Preliminary themes to initial research question	Missing information about the case Missing information about the risk for exacerbation of apical periodontitis Difficulty assessing the risk in individual cases Uncertainty leading to health economic considerations A lack of personal knowledge or experience The advantages of being an expert when conducting risk assessment
New research question focusing on experience	How did the students reflect on clinical experience in relation to uncertainty when assessing the risk for exacerbation of apical periodontitis in root‐filled teeth?
Preliminary themes focusing on experience	A lack of personal knowledge or experience The advantages of being an expert when conducting risk assessment

The students' reflections regarding experience were connected to prompt 6 (about expert vs. student reasoning) and in some cases to prompt 7 (about changing strategies if proved wrong; Table 1). The texts about experience were between 2 and 204 words long with a median of 42 words. After step 4 of the STC, describing categories from the condensates, all the reflections were re‐read by J.B. and H.F. to ensure that all themes, codes, subgroups, and categories were representative, and some adjustments were made. To ensure trustworthiness, the data was analysed separately by the authors in each step and in case of disagreement, there was a discussion to widen the analysis. The citations chosen to illustrate the subgroups were translated from Swedish to English by an authorised translator with professional experience from working at a dental school and translated back to Swedish to verify that the translation was correct. The students were not requested to comment on the findings. The COREQ (COnsolidated criteria for REporting Qualitative research) checklist was used for the preparation of this manuscript (Tong et al. 2007).

## 3 Results

Two themes were identified during the qualitative analysis of the reflections: (1) *the meaning of clinical experience*, which was divided into five subgroups, and (2) *differences and similarities*, divided into four subgroups (Table 4).

**TABLE 4:** Themes and subgroups identified in the students' reflections about experience and lack of experience.

Themes	Subgroups
1. The meaning of clinical experience	1.1 Encountering several similar cases leads to knowledge 1.2 With experience comes knowledge of risk factors 1.3 Experts have the answers 1.4 Experts are more prone to wait with intervention 1.5 The ability to perform treatment affects the decision to intervene
2. Differences and similarities	2.1 A lack of primary experience makes it more difficult to assess the risk 2.2 Level of experience influence assessment 2.3 Inexperienced dentists may over diagnose and make too many examinations 2.4 Relating to the same risk factors

### 3.1 Theme 1: The Meaning of Clinical Experience

#### 3.1.1 Encountering Several Similar Cases Leads to Knowledge

Several of the participants discussed what clinical experience signifies. They described an expert as someone who has encountered and treated many similar cases and therefore can assess the risk faster and with greater certainty.

Experience was described as leading to experts being able to recognise specific types of apical destructions and know their probable course of development. One participant described that by having treated similar cases and having seen if they healed, an expert could have received more information about why it looks like it does on the radiograph.

The more cases one sees the better one becomes at recognizing typical lesions and how they are likely to progress. In this case one could previously have seen something similar and after carrying out treatment would have acquired more information about the condition and why it appears the way it does on the xray. One might have done a root filling and observed that the lesion then heals. (Participant 40)

#### 3.1.2 With Experience Comes Knowledge of Risk Factors

Several participants described that experts have more knowledge about risk factors since they have encountered more clinical cases. It was also mentioned that experts are more knowledgeable, have greater knowledge about statistics for risk assessment, have a greater knowledge bank, and perhaps their own checklist of risk factors.

Some participants also described how an expert can judge what information is important with more ease and is better at understanding situations and looking at the whole picture and is better at relating the risk to the patient's general health. They also wrote that experts are better at assessing which risk factors affect the speed of progression of apical periodontitis and that an expert feels more comfortable when assessing the risk for exacerbation.

An expert in this field would probably reason with greater experience and therefore have more confidence about which conditions and factors are involved in exacerbation of this condition. (Participant 44)

#### 3.1.3 Experts Have the Answers

Furthermore, the participants described that experts have seen how and in which patients' exacerbations usually occur and can distinguish a pattern, which means that they have a better sense of how it looks in practice. Participants also wrote that experts are better at risk assessment because of their experience of having seen and followed root‐filled teeth. They wrote that experts have been able to see the outcome and have what one participant described as ‘the answers’, something that the participant felt they were missing.

The experts may be just as uncertain as we are. But I WANT to believe that they have more experience and that by taking into account the x‐ray, the patient's health, the quality of the restoration and the quality of the root filling, they can determine whether there is a risk of an acute condition developing within the next 4–5 years. The difference is that they have experience. They look at the same factors as I do to determine risk but they already have the answers. They have assessed many such cases and followed the outcome. We don't have that frame of reference! (Participant 15)

One participant described that experts can draw conclusions from their cases as if they were a published clinical study.

I believe that the expert uses the same reasoning as I do, but at the same time I think that because of experience the expert can take more factors into account in their reasoning. Even when one as an expert reasons in such as way that cases one has encountered function as one's own clinical study, whereby one perhaps had to choose between treating or not treating, in the same type of cases for some reason (e.g., one patient may be ready to accept treatment, another may not) and one has seen the outcome of this. (Participant 45)

#### 3.1.4 Experts Are More Prone to Wait With Intervention

Some participants wrote that based on experience, an expert is better able to determine the appropriate treatment and the level of difficulty. They also wrote that an expert could feel more certain when assessing in which cases it is appropriate to initiate treatment and in which cases it is appropriate to wait and see. Some participants also described that perhaps an expert would choose to wait with treatment and dare to remain composed in their decision‐making. One participant thought experience would have shown experts that it is usually safe to wait.

Perhaps a more experienced clinician would be more likely to wait and see than an inexperienced clinician. According to lecturers at dental school one elects to undertreat in many cases and this is based probably on the experience that the risks of waiting are often not as great as one has thought. (Participant 26)

#### 3.1.5 The Ability to Perform Treatment Affects the Decision to Intervene

A number of participants described that experts believe they can perform a better root canal treatment than the patient's previous dentist. In contrast to the participants who thought that an expert rather than an inexperienced dentist would wait and see, one participant wrote that the decision to perform a re‐treatment of a root filled tooth is affected by the fact that experts believe that they can redo the root filling and improve it, and that this makes it more likely that experts will decide on re‐treatment. Other participants mentioned that an expert can offer other treatment methods than the participants.

I believe that an expert has greater clinical experience and assesses the risk for exacerbation differently. It is more likely that an expert would redo a root filling on pathological or technical indications because they consider that they can do this much better than previous dentists. A newly qualified dentist might be more willing to wait and see if there are no obvious risks, because of the patient's general health, for example. (Participant 19)

### 3.2 Theme 2: Differences and Similarities

#### 3.2.1 A Lack of Primary Experience Makes It More Difficult to Assess the Risk

Some participants wrote that they lacked the experience of having seen or treated similar cases. They thought that they would feel more certain and that it would be easier to reason and assess the risk when they had more clinical experience. One participant mentioned that a novice could assess the risk as higher due to this lack of experience.

But with time, when I have treated lots of such cases, perhaps I will feel more confident of what is right for the individual patient. (Participant 24)

I haven't much experience of cases of exacerbation of apical periodontitis (in theory, yes, but in practice, no) and I therefore believe that when I am faced with such cases I will learn to reason and assess the risk in a better way. (Participant 9)

One participant pointed out that just because the experts, thanks to their experience, would do a better risk assessment, the participant's assessment does not have to be poor.

#### 3.2.2 Level of Experience Influence Assessment

Several participants believed that an expert's clinical experience, and the fact that the expert had encountered many more clinical cases, would produce a different assessment than their own in several ways. They mentioned that an expert could make a different assessment regarding risk and defence factors, cost–benefit considerations, factors relevant to the initiation and progression of periapical disease, and the quality of the root filling. One participant speculated that an expert's assessment could differ but pointed out that more research is still needed in the area.

They have seen many more cases than I have and have greater clinical experience and therefore their assessment could be different. However, there is a need for more scientific research into the condition. (Participant 28)

#### 3.2.3 Inexperienced Dentists May Overdiagnose and Make Too Many Examinations

Some participants expressed uncertainty about diagnostics compared to an expert. One participant expressed uncertainty about interpreting radiographs as a novice and thought that a more experienced dentist would find it easier to detect differences in the size of the lesion. Another participant thought that a new dentist would perform more examinations compared to an experienced dentist.

But it's clear there is a difference in reasoning: because I am completely new I might perhaps do a more extensive examination than an experienced dentist who is more familiar with such cases and can see just which examinations are required for this particular patient. (Participant 27)

One participant wrote that an inexperienced dentist's fear of exacerbation of apical periodontitis could lead to a higher risk of over diagnosing.

As a general dental practitioner without much experience I would probably be more likely to overdiagnose because of concern that the condition might exacerbate. It is just my ignorance about exacerbation which would result in my under/overdiagnosis of this case. Just now I have the knowledge that I can be guided by earlier radiographs to determine whether there has been healing or not. If there has been healing then I consider that the risk of exacerbation is low and i would therefore not initiate treatment. If I start treatment because i am uncertain, that wouldn't feel right either, it will be a long expensive procedure for the patient and at the same time we are using a lot of the clinic's resources. I need more knowledge in this field in order to consider changing strategy just now as I have nothing more to go on than previous radiographs, fillings and case notes. (Participant 44)

#### 3.2.4 Relating to the Same Risk Factors

Some participants had an ambiguous view of the connection between the assessment of risk factors and experience, where several participants wrote that an expert and a novice take the same risk factors into consideration, but at the same time pointed out that experience could possibly lead to a different assessment of these risk factors.

Attack and defense factors are the same for all patients and one could possibly assess these differently in light of one's experiences and expertise. (Participant 16)

One participant believed that the assessment of the quality of the root filling would differ between an expert and a novice, but not the assessment of other risk factors.

The experts have of course greater experience/competence and on this basis can express an opinion about the risk. Their assessment of the root filling (quality) is probably better than mine. However I do not believe that our assessment would differ when it comes to other factors which I take into account in assessing the risk. (Participant 30)

Several participants wrote that the theoretical reasoning about the risk assessment, based on information about the patient and about the case, would not differ between an experienced dentist and a novice. One participant wrote that they had such vague information about this case that an expert and a novice would reason similarly. Another participant described how it is impossible for anyone to determine the risk.

## 4 Discussion

The idea was that by letting students reflect on simulated cases involving uncertainty, they would learn how to deal with uncertain cases in the future (Scott et al. 2020). The content of the reflections indicated that the students believed that experience leads to certainty. For example, one student wrote that in the future, when they would have more experience, they would feel more certain about what would be the best strategy for a specific patient with a root‐filled tooth apical periodontitis.

The lack of scientific evidence about various factors involved in assessing the risk for exacerbation of apical periodontitis includes information about the frequency of exacerbations and spread of infection, the interaction between the microorganisms involved and the host defence, and the role of overall health (Pigg et al. 2022). In addition, this scientific uncertainty is further complicated by the more general uncertainty always inherent in medical or dental treatment involving a patient such as the unknown chance of a specific outcome or the probability of disease progression in this particular individual (Hofmann 2021). Taken together, this makes it impossible for anyone—novice or expert—to be truly certain about the risk for exacerbation of apical periodontitis in a root‐filled tooth with apical periodontitis.

Some of the students mentioned that they had only theoretical and no clinical experience of assessing the risk of exacerbation of root‐filled teeth with apical periodontitis. The value of clinical hands‐on experience was corroborated in another qualitative study analysing dental students' reflections on the experience of conscious sedation, where one of three identified themes in the qualitative analysis was ‘confidence through experience’. The students considered hands‐on experience the most valuable factor for confidence (Crowe and Woolley 2022).

Another explanation for students' belief that experience would lead to certainty was that many students believed that an expert could draw conclusions about how new cases would develop based on their personal experience of previous treatments. One student wrote that experts could use their own cases as scientific evidence as if they were clinical studies. For this to be possible at all, root‐filled teeth must be followed up after treatment, which is not always the case (Malmberg et al. 2020). Dentists do not seem to agree that their own treated cases can be seen as a source of evidence. A study on Swedish dentists showed that they did not consider personal experience comparable to scientific evidence and that it lacked the systematic approach of scientific evidence (Sahlin et al. 2020). This suggests that the students may not have a clear understanding of, first, what kind of feedback the dentist gets after treatment, and second, the scientific value of personal experience.

Some students also wrote that experts are more prone to wait with intervention, and that they are more comfortable with risk assessment. There were signs in the texts that some students had been influenced by their lecturers' opinions about overtreatment and undertreatment. One student wrote that according to a lecturer, undertreatment was desirable in many cases and that the lecturer had probably seen that there was not as much risk in waiting with treatment as one could have expected. This belief that experts would be more prone to wait with treatment was not shared by all students. Some students reflected on how the skill to perform re‐treatment would affect the decision to intervene, concluding that an expert would be more prone to decide to retreat the tooth compared to a novice. This reflection was supported by the findings in a study exploring endodontic education level and re‐treatment decision. In a case with a root‐filled tooth with an incomplete root canal filling, lacking coronal restoration, and with a 5 mm periapical lesion, 76.2% of the endodontists preferred re‐treatment over extraction or no treatment compared to 22.4% of the undergraduate students. The authors reflected that the students in their study, similar to the present study, only had experience of a few re‐treatment decisions and mostly responded by using their theoretical knowledge (Alim‐Uysal et al. 2021).

Some students in the present study also stated that inexperienced dentists may overdiagnose and perform too many examinations. In another study involving medical doctors, a strong correlation was found between experience and lower risk aversion. Less experienced doctors chose more risk averse treatment strategies which included ordering more tests, offering more treatments, and referring more frequently. This was explained as a lower tolerance of uncertainty among unexperienced doctors (Lawton et al. 2019), which suggests that the difference lies in the comfort with uncertainty rather than in amount and quality of knowledge. Dental experts are perhaps equally uncertain and not more knowledgeable about the probable outcome of the treatment of apical periodontitis. Experts would then simply be more comfortable with making a treatment decision under uncertain conditions, which runs counter to what the students in this present study thought.

The value of written reflections as data for qualitative analysis is somewhat limited since the texts depended on the student's willingness to develop their thoughts in writing (Malterud and Midenstrand 2009). However, several other studies have used students' reflections as a base for qualitative studies in dentistry (Crowe and Woolley 2022; Boyd 2002; Jones et al. 2016).

Data from written material may not give as in‐depth understanding as data from interviews and should be interpreted with some caution. The STC used for analysing the data has been described as a pragmatic method of qualitative analysis suitable for novices and for different analyses including the analysis of written text (Malterud 2012) and was considered well suited for this study. The analysis led to the identification of two themes with total nine subgroups, which allowed different nuances of the themes to be reflected.

Since the student reflections demonstrate that students believe they will be certain as soon as they have more experience, the findings from this study may have implications for future dental education. Firstly, in dental education the recognition and management of uncertainty needs to be acknowledged in the curriculum (Shuler and von Bergmann 2021). Secondly, there may also be a need for faculty development, such as teaching educators appropriate courses of action of working with uncertainty (Moffett et al. 2022). A way to move forward could be for experienced clinicians to demonstrate for novices how they deal with feelings of uncertainty and show the strategies they use (Lawton et al. 2019). Thirdly, in a more subject‐specific perspective, the reported lack of primary experience in risk assessment of root‐filled teeth with apical periodontitis is an important finding and highlights a need for more clinical training in this task.

Lastly, one student specifically mentioned that clinical experience would lead to a better understanding of biology. On the contrary, a well‐known problem in medical education is that the knowledge in basic sciences gained in the early years of education tend to be forgotten in a clinical context later. For a better understanding, basic science concepts need to be connected to clinical decision‐making (Ganguly et al. 2019). Barriers to successful integration between basic and clinical science was reported by faculty members of a dental school in Texas, USA to include lack of time for preparation of the integrated courses, limited possibility for basic scientists and clinicians to interact, and lack of insight into what was being taught in the other curriculum (van der Hoeven et al. 2018). Thus, it seems that clinician educators and basic scientists must work together and be given time by the faculty to improve this interaction. This study focused on students facing a problem in endodontics, namely risk assessment in association with a root‐filled tooth with symptom‐free apical periodontitis. Their reflections revealed interesting insights into novices' beliefs and reasoning about experience and certainty, which could conceivably relate also to other situations with inherent complex uncertainty, in dentistry as well as in other disciplines. The conclusion is that the students expressed a strong belief that with clinical experience comes certainty and that clinical experience would help in making risk assessment even when the scientific evidence is lacking.

## Author Contributions

J.B. designed the reflection exercise, collected the data, analysed the text and wrote the first draft. H.F., N.V. and M.P. participated in designing the reflection exercise and contributed to conducting the analysis, writing, editing and performing a critical review. All authors gave their final approval and agreed to be accountable for all aspects of the work.

## Ethics Statement

The Regional Ethical Review Authority determined that the study did not require a permit according to Swedish law, but issued an advisory statement approving the study, Dnr 2019‐02441. The research has been conducted in full accordance with ethical principles, including the World Medical Association Declaration of Helsinki (version 2013).

## Consent

The authors have nothing to report.

## Conflicts of Interest

The authors declare no conflicts of interest.

## Data Availability

The data that support the findings of this study are available from the corresponding author upon reasonable request.
